# Histopathological and Molecular Characterization of Amlodipine-Induced Gingival Enlargement: Insights into Fibrotic Mechanisms

**DOI:** 10.3390/ph19010045

**Published:** 2025-12-24

**Authors:** Jana Mojsilović, Marina Kostić, Sanja Vujović Ristić, Momir Stevanović, Milovan Stević, Sanja Knežević, Nemanja Jovičić

**Affiliations:** 1 Department of Dentistry, Faculty of Medical Sciences, University of Kragujevac, Svetozara Markovića 69, 34000 Kragujevac, Serbia; jana.desnica@gmail.com (J.M.); momirstevanovic7@gmail.com (M.S.); milovan_stevic@yahoo.com (M.S.); 2 Department of Pharmacology and Toxicology, Faculty of Medical Sciences, University of Kragujevac, Svetozara Markovića 69, 34000 Kragujevac, Serbia; 3 Center for Research on Harmful Effects of Biological and Chemical Hazards, Faculty of Medical Sciences, University of Kragujevac, Svetozara Markovića 69, 34000 Kragujevac, Serbia; 4 Department of Pediatrics, Faculty of Medical Sciences, University of Kragujevac, Svetozara Markovića 69, 34000 Kragujevac, Serbia; sanjaknez1980@yahoo.com; 5 Department of Histology and Embryology, Faculty of Medical Sciences, University of Kragujevac, Svetozara Markovića 69, 34000 Kragujevac, Serbia; nemanjajovicic.kg@gmail.com

**Keywords:** amlodipine, gingival enlargement, extracellular matrix, IL-13, α-SMA, MMP-1, procollagen, fibrosis, histopathology, real-time PCR

## Abstract

**Background/Objectives**: Amlodipine, a widely prescribed calcium channel blocker, has been associated with gingival enlargement, yet the mechanisms underlying this adverse effect remain unclear. The present study aimed to explore molecular and histopathological factors potentially contributing to gingival changes in patients receiving amlodipine therapy, with a particular focus on molecules implicated in extracellular matrix turnover and tissue remodeling. **Methods**: The study included three groups of participants: patients with amlodipine-induced gingival enlargement, patients with gingival enlargement of inflammatory origin, and amlodipine-treated patients without gingival overgrowth. Gingival tissue samples were analyzed using hematoxylin-eosin staining to assess inflammatory changes and general tissue architecture, and Picrosirius Red staining to visualize collagen fibers. Relative gene expression of alpha-smooth muscle actin (α-SMA), IL-13, MMP-1, and procollagen was determined by real-time PCR, while collagen content was quantified using ImageJ software. **Results**: Histopathological evaluation revealed a less pronounced inflammatory response in amlodipine-related gingival enlargement compared to those who did not use amlodipine. The highest expression of α-SMA was detected in patients who did not receive amlodipine, whereas IL-13 and procollagen expression were markedly elevated in the amlodipine-induced group compared to others. MMP-1 expression was significantly lower in amlodipine-treated patients relative to those who did not use amlodipine, suggesting impaired collagen degradation. These findings, together with our previous results indicating enhanced expression of profibrotic mediators, suggest that altered extracellular matrix metabolism is potentially dominant in this condition. **Conclusions**: Amlodipine-induced gingival enlargement appears to involve a multifactorial process characterized by a prominent fibrotic component, reduced matrix degradation, and secondary inflammation.

## 1. Introduction

Drug-induced gingival enlargement is a recognized and widespread adverse effect, most commonly associated with the use of phenytoin, cyclosporine, and nifedipine, a first-generation calcium channel blocker (CCB) [1]. However, amlodipine, a third-generation CCB, has also been increasingly reported to cause this adverse reaction, although fewer scientific data are available regarding its underlying mechanisms and clinical use [2]. Drug-induced gingival enlargement is characterized by an increase in gingival volume, typically affecting the papillary or marginal tissues. It is more frequently observed in the anterior than in the posterior region, and on the buccal rather than lingual or palatal surfaces. Depending on its severity, this condition may compromise aesthetics and function, while also favoring bacterial biofilm accumulation, which contributes to periodontal pathology [3]. Recent histological investigations have demonstrated that enlarged gingival tissue differs from healthy gingiva, most notably by increased fibrosis and elongated rete pegs. The histological characteristics of drug-induced gingival enlargement (DIGE) also include thickening of the epithelium, increased numbers of fibroblasts and blood vessels in the connective tissue matrix, and accumulation of ground substance. Variations in the degree of fibrosis have also been reported among drug-induced cases, based on semiquantitative histological assessments. More recent findings indicate disruption of the basal membrane, characterized by discontinuous collagen type IV and decreased laminin 5 expression [4,5].

The mechanism of CCBs-induced gingival enlargement is not fully understood. However, CCBs can disrupt fibroblast calcium metabolism, impairing collagenase activity and leading to extracellular matrix (ECM) accumulation. Gingival tissue undergoes continuous cycles of injury and repair, regulated mainly by cytokines and chemokines. Among these, transforming growth factor-β1 (TGF-β1) promotes fibroblast recruitment and ECM protein synthesis, while connective tissue growth factor (CTGF) acts downstream of TGF-β1, amplifying fibrotic responses [6,7]. TGF-β1 is also a key inducer of epithelial–mesenchymal transition (EMT), a process in which epithelial cells migrate into the connective tissue and acquire fibroblast-like characteristics. During pathological EMT, myofibroblasts emerge and are characterized by de novo expression of alpha-smooth muscle actin (α-SMA), which is widely used as a molecular marker associated with fibrosis [5]. In addition to TGF-β1, other cytokines have been implicated in the regulation of fibrotic processes. Interleukin-13 (IL-13) has been identified as a profibrotic cytokine involved in several fibroproliferative disorders, including pulmonary fibrosis, hepatic cirrhosis, myocardial and renal fibrosis, and pathological scarring [8]. Its activity promotes collagen synthesis and accumulation by fibroblasts, and recent evidence indicates that combined inhibition of IL-13 and TGF-β signaling more effectively suppresses fibrotic pathways than TGF-β inhibition alone [9]. In parallel, matrix metalloproteinase-1 (MMP-1), a key enzyme responsible for collagen degradation and extracellular matrix remodeling, has been shown to play an opposing yet complementary role. Reduced MMP-1 expression, reported in DIGE, particularly in patients treated with cyclosporine, contributes to impaired collagen turnover and excessive connective tissue deposition. Conversely, increased MMP-1 activity observed in inflammatory periodontal disease suggests that the imbalance between matrix synthesis and degradation may be a crucial determinant of gingival fibrosis [10,11].

This study aimed to further elucidate the mechanisms underlying amlodipine-induced gingival enlargement through an integrated histopathological and molecular analysis. We assessed fibrosis, collagen deposition, as well as the relative gene expressions of α-SMA, IL-13, MMP-1, and procollagen, to better define their roles in gingival tissue remodeling and fibrogenic activity. In contrast to our previous investigation conducted in the same patient cohort [12], which focused primarily on oxidative stress and cytokine- and growth factor-related alterations, the present study addressed structural and matrix-related changes in gingival tissue. By combining histological assessment with the analysis of relative gene expression of selected molecular markers linked to extracellular matrix turnover and fibroblast-related pathways, this study provided complementary and incremental information on the tissue-level features of amlodipine-induced gingival overgrowth.

## 2. Results

The study included gingival tissue samples from 24 patients, 7 males and 17 females. Among them, 8 patients belonged to the group with amlodipine-induced gingival enlargement (A+GE+), 8 patients were classified into the group with gingival enlargement of other, mainly inflammatory, etiologies (A−GE+), while the remaining 8 patients, who were receiving chronic amlodipine therapy, showed no clinical signs of gingival enlargement (A+GE−). Our previous study found no significant differences in sociodemographic characteristics among the patients [12]. Table 1 presents the distribution of the estimated indices across all three patient groups. No statistically significant differences were observed among the groups for the estimated parameters, with the exception of the Papillae Bleeding Index, which was highest in patients with inflammatory gingival enlargement.

Table 2 presents the duration of amlodipine therapy prior to sampling in both groups. No statistically significant difference was observed between patients with and without this adverse effect.

### 2.1. Histopathological Analysis of Gingival Tissue Architecture

**Figure 1:**
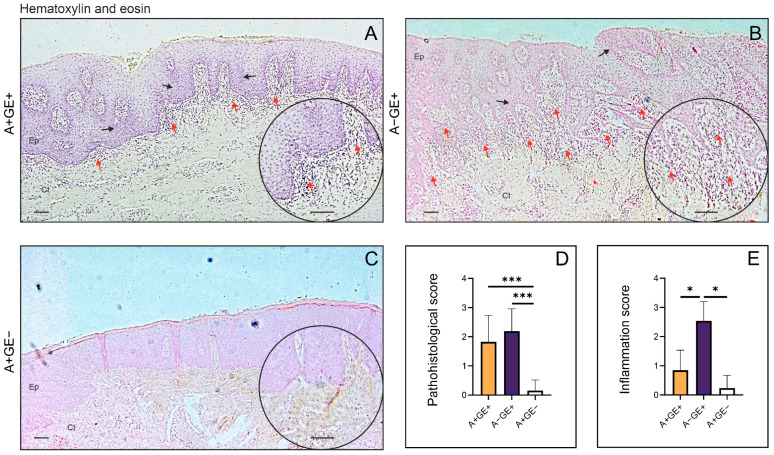
Histopathological analysis of the gingival tissue. Hematoxylin and eosin staining; photomicrographs; rectangle ×20, circle ×40; (**A**) Patients receiving amlodipine with gingival enlargement; (**B**) Patients with gingival enlargement not receiving amlodipine; (**C**) Patients receiving amlodipine without gingival enlargement; (**D**) Pathohistological score in gingival tissue; (**E**) Inflammation score in gingival tissue; mean ± SE (* *p* < 0.05; *** *p* < 0.001; Ep—epithelium; Ct—connective tissue; Black arrows—epithelial hyperplasia; Red arrows—inflammatory cells; Scale bar = 100 µm).

Histopathological analysis of the isolated gingival tissue was performed on samples fixed in formalin and embedded in paraffin blocks. Using hematoxylin and eosin staining, gingival tissue from patients with gingival enlargement showed extensive epithelial hyperplasia and acanthosis, as well as pronounced epithelial ridges extending deeply between the connective tissue papillae, which is a characteristic feature of this type of lesion (Figure 1A,B). In patients receiving amlodipine therapy who exhibited pronounced gingival enlargement, focal accumulations of mononuclear inflammatory cells were observed within the gingival connective tissue. In contrast, gingival tissue from patients with gingival enlargement unrelated to amlodipine use contained a large number of inflammatory cells, organized either in focal clusters or diffusely distributed mononuclear infiltrates (Figure 1A). Pathohistological score in gingival tissue was significantly higher in groups with gingival enlargement (Figure 1D). Further, the inflammatory score used for analysis showed a statistically significant increase in inflammation in the gingival tissue of patients with non-amlodipine-associated gingival enlargement compared to the other examined groups (Figure 1E).

**Figure 2:**
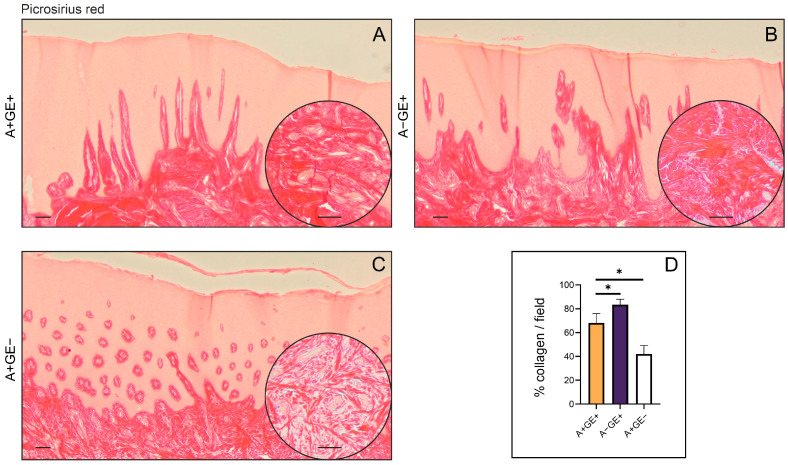
Histopathological analysis of the architecture of the gingival connective tissue. Picrosirius Red staining; representative photomicrographs; rectangle ×20, circle ×40. (**A**) Patients receiving amlodipine with gingival enlargement. (**B**) Patients with gingival enlargement not receiving amlodipine. (**C**) Patients receiving amlodipine without gingival enlargement. (**D**) Semiquantitative analysis of collagen fiber distribution in photomicrographs; mean ± SD (* *p* < 0.05). Scale bar = 100 µm.

Using the selective histochemical Picrosirius red technique, the architecture of the gingival connective tissue and the organization of collagen fibers were analyzed. In these samples, elongated connective tissue papillae densely packed with bundles of collagen fibers were observed in patients with gingival enlargement (Figure 2A,B). In the deeper layers of the gingival connective tissue, a high density of thick collagen fiber bundles, as well as an increased number of fibroblasts and blood vessels, was noted compared to tissue from patients without gingival enlargement (Figure 2C). Semiquantitative analysis of the photomicrographs revealed that the percentage of the field occupied by collagen fibers was significantly higher in patients with gingival enlargement unrelated to amlodipine use compared to the other groups (Figure 2D).

### 2.2. Molecular Analysis: Gene Expression Assessment by RT-PCR

RT-PCR analysis was conducted to evaluate the relative gene expression levels of key fibrogenic markers, including α-SMA, procollagen, IL-13, and MMP-1, in gingival tissue samples from all study groups. The results revealed distinct expression patterns among patients with gingival overgrowth of different etiologies and those undergoing chronic amlodipine therapy.

**Figure 3:**
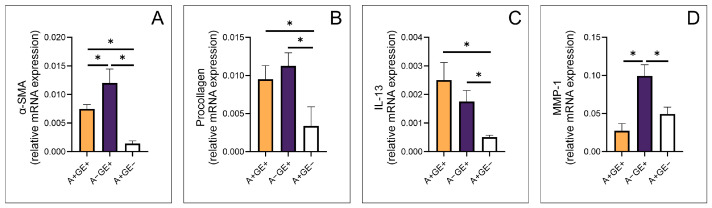
Molecular alterations in gingival tissue (relative gene expression; three equal groups, n = 24; A+GE+—amlodipine with gingival enlargement; A−GE+—gingival enlargement without amlodipine; A+GE−—amlodipine without gingival enlargement); (**A**) α-SMA; (**B**) Procollagen; (**C**) IL-13; (**D**) MMP-1; the values are presented as mean ± SD (* *p* < 0.05).

The relative expression of the α-SMA gene was significantly different in patients with gingival overgrowth of inflammatory etiology compared to both groups receiving amlodipine. Additionally, patients with amlodipine-induced gingival overgrowth exhibited significantly higher α-SMA relative gene expression than those on chronic amlodipine therapy without gingival enlargement (Figure 3A).

Patients with amlodipine-induced gingival enlargement demonstrated significantly increased procollagen gene expression compared to those on long-term amlodipine therapy without gingival enlargement. Furthermore, a statistically significant difference in procollagen gene expression was observed between these patients and those with gingival overgrowth of other etiologies (Figure 3B).

Patients with amlodipine-induced gingival overgrowth exhibited significantly higher IL-13 gene expression compared to amlodipine-treated patients without gingival enlargement. Similarly, patients with gingival overgrowth of other etiologies also showed significantly higher IL-13 gene expression than those without this adverse effect (Figure 3C).

MMP-1 gene expression was significantly elevated in patients with gingival overgrowth of non-amlodipine (inflammatory) etiology compared to both groups receiving chronic amlodipine therapy (Figure 3D).

## 3. Discussion

This study aimed to investigate the fibrotic component of amlodipine-induced gingival enlargement and used histopathological scores to determine the presence of an inflammatory component and to assess the architecture of gingival connective tissue in patients with amlodipine-induced gingival enlargement. The study included three groups: (1) patients with amlodipine-induced gingival enlargement, (2) patients with gingival changes in inflammatory origin who do not take amlodipine, and (3) a negative control group consisting of patients on chronic amlodipine therapy who do not exhibit gingival enlargement. This design aimed to identify significant differences among groups, thereby exploring potential mechanisms associated with the occurrence and pathogenesis of this condition, which is still not sufficiently elucidated.

Based on histopathological scoring, inflammation was significantly more pronounced in patients without gingival enlargement than in both amlodipine groups, regardless of whether gingival overgrowth was present. These findings are consistent with our previous research on this topic, suggesting that while inflammation may be involved in the overall tissue response, it may not represent the dominant driving factor in the development of amlodipine-induced gingival enlargement [12]. Furthermore, earlier studies on human samples reported no significant difference in the histopathological inflammation scores between patients with gingival enlargement of inflammatory origin and those with amlodipine-induced gingival enlargement [4]. Although our findings differ in that respect, it should be emphasized that our study also included patients treated with amlodipine who did not develop gingival enlargement. Given that no significant difference in inflammation was observed between these two amlodipine-treated groups, the overall results support an association between gingival enlargement and mechanisms beyond inflammation alone, rather than indicating a primary inflammatory etiology.

Previous studies have indicated that a fibrotic component likely exists in drug-induced gingival enlargement, though the exact mechanisms remain unclear. Fibroblasts are thought to play a central role, given their function in producing and degrading ECM proteins. Fibroblasts from phenytoin-induced gingival enlargement reportedly show higher proliferation and collagen synthesis than those from healthy gingiva, suggesting a possible selective pressure favoring a more fibrogenic subpopulation. Previous ultrastructural studies indicated the presence of myofibroblast-like cells. Myofibroblasts, typically characterized by α-SMA expression, have been proposed as contributors to ECM deposition during tissue remodeling. Under normal conditions, such as wound healing, these cells usually undergo apoptosis once repair is complete. When this process fails, their persistence may promote excessive ECM accumulation and potentially lead to pathological fibrosis [13,14,15]. In the present study, α-SMA was assessed at the relative gene expression level. The highest α-SMA gene expression was observed in the second group (patients with inflammatory gingival enlargement). However, a significant difference was also noted between patients who developed gingival enlargement and those who did not, suggesting differential regulation of ACTA2 transcription rather than providing direct evidence of increased myofibroblast abundance. This may be explained by acute tissue damage and a more intense defense response. In contrast, amlodipine-induced gingival enlargement likely lacks such acute injury. Moreover, recent studies have indicated a potential link between periodontitis and EMT, which could help explain why α-SMA gene expression was the highest in the group with inflammatory gingival enlargement [16].

Procollagen gene expression levels were notably higher in patients with gingival enlargement compared to those without this adverse effect. This observation is consistent with the concept that procollagen expression may reflect ongoing fibrotic activity, as this molecule has been proposed as a potential biomarker of fibrosis [17,18,19], suggesting that procollagen gene expression is more likely to be elevated in patients with existing gingival enlargement.

Evidence from previous studies suggests that IL-13 may contribute to abnormal fibroproliferative activity. Interestingly, a recent report demonstrated that in fibrotic conditions, simultaneous inhibition of both IL-13 and TGF-β1 signaling completely attenuated the fibrotic mechanism compared with inhibition of TGF-β1 alone. Functionally, the IL-13 signaling pathway may activate fibroblasts to produce extracellular matrix components and other related factors necessary for collagen fibrogenesis. IL-13 has also been reported to induce TGF-β1 expression, acting primarily on fibroblasts and endothelial cells, which results in increased collagen and matrix synthesis [9,20]. In our study, higher levels of IL-13 gene expression were observed in the group of patients with amlodipine-induced gingival enlargement compared to those without this adverse effect. These analyses were conducted on the same tissue samples from the previously studied patient cohort, in which elevated TGF-β1 relative gene expression was also observed in our previous research [12]. The concordance between IL-13 relative gene expression and our earlier findings may point toward a potential association between these pathways in the fibrotic component of gingival enlargement [12]. Importantly, differences in molecular pathways between drug-induced and inflammatory gingival fibroses have been reported, suggesting that cytokines such as IL-13 may be regulated in a context-dependent manner [21]. Therefore, the observed increase in IL-13 relative gene expression levels should be interpreted within this heterogeneous context. Our results may indicate that the fibrotic component of gingival enlargement may involve a network comprising IL-13 and previously identified pathways for TGF-β1, CTGF, and oxidative stress. Oxidative stress has been reported to potentially enhance TGF-β1 activation, suggesting that synergistic interactions among these mechanisms may have occurred [12]. Furthermore, prior studies have shown that increased oxidative stress enhances TGF-β1 activation [22,23], supporting the hypothesis that these pathways may act synergistically. Collectively, the current and our previous findings support the possibility that IL-13, in conjunction with TGF-β1/CTGF signaling and oxidative stress, may be involved in the molecular framework underlying amlodipine-induced gingival enlargement. While further research is necessary to clarify these interactions, targeting IL-13 signaling along with TGF-β1 and oxidative stress pathways may offer a promising strategy for the prevention and management of this condition.

MMP-1 plays a crucial role in initiating extracellular matrix degradation and, with other matrix MMPs, facilitates the breakdown of collagen. Previous studies have reported a significant reduction in MMP-1 expression in overgrown gingival tissues of organ transplant recipients receiving cyclosporine A. Further investigations suggest that cyclosporine A suppresses both the synthesis and enzymatic activity of MMP-1 in gingival fibroblasts, which may contribute to gingival enlargement. Conversely, increased levels of MMP-1 mRNA have been identified in gingival tissues from patients with periodontitis, indicating a distinct regulatory mechanism in inflammatory conditions [10,24,25]. In our study, even though amlodipine likely does not share the same mechanism of gingival enlargement as cyclosporine, the results are consistent with previous research on drug-induced gingival overgrowth associated with this medication [26]. Patients treated with amlodipine, regardless of whether they developed gingival enlargement, exhibited significantly lower MMP-1 gene expression levels compared to those with gingival overgrowth of inflammatory etiology. This contrast further supports the notion that drug-induced and inflammatory gingival enlargement may involve partially divergent molecular pathways. Furthermore, previous findings suggest that amlodipine, unlike nifedipine, may directly suppress MMP-1 expression in IL-1β-stimulated endothelial cells, thereby reducing collagenolytic activity [27]. Our results are in line with the possibility that a similar mechanism may operate in gingival tissue, where amlodipine could contribute to decreased MMP-1 expression and subsequent extracellular matrix accumulation. Previous studies indicated that gingival enlargement associated with CCBs is mediated by distinct molecular mechanisms depending on the specific agent [28]. Nifedipine is reported to induce gingival overgrowth more frequently than amlodipine, a difference attributed to variations in pharmacokinetic properties, tissue distribution, and the cellular responses of gingival fibroblasts. In contrast, diltiazem is less consistently associated with gingival enlargement, with available data suggesting weaker or more variable effects on gingival fibroblast behavior compared to dihydropyridine CCBs [21,28]. The reduced MMP-1 relative gene expression observed in amlodipine-treated patients in the present study may therefore reflect a drug-specific modulation of matrix degradation pathways. These findings further support the concept that calcium channel blocker-associated gingival enlargement is a potentially heterogeneous condition with agent-dependent molecular characteristics.

## 4. Materials and Methods

A cross-sectional study was conducted in routine clinical practice to determine the pathohistological characteristics and mechanisms underlying gingival enlargement in patients receiving chronic amlodipine therapy.

### 4.1. Study Population, Inclusion and Exclusion Criteria

Tissue samples were collected from adult patients of both sexes diagnosed with hypertension. The inclusion criteria were established as follows: participants were required to have undergone treatment with amlodipine for a minimum duration of six months, retain all anterior teeth (at least 12 in total), and demonstrate a clinical necessity for surgical intervention. This surgical need could arise from conditions such as gingival overgrowth or the necessity for additional oral or periodontal surgical procedures, provided that the sampling of gingival tissue was both clinically justified and ethically permissible.

The exclusion criteria for the study included the following: individuals who were pregnant or breastfeeding, those who had undergone antibiotic therapy within the three months preceding enrollment, patients with uncontrolled systemic diseases, and smokers. Additionally, participants taking other medications known to cause gingival enlargement, such as cyclosporine A or phenytoin, were also excluded.

### 4.2. Sample Size

The sample size was determined using the G*Power program (version 3.1.9.7), with the ANOVA test (fixed effects, omnibus, one-way), based on data from a similar study [4]. The analysis determined that 7 patients per group should be included, but for this research, 8 patients per group were ultimately included.

### 4.3. Procedure

All participants gave their written informed consent before being included in the study. They first received complete information about the study objectives and procedures. The study protocol received approval from the Ethics Committees of the University Clinical Center Kragujevac (decision number: 01-22-390; approval date: 4 November 2022), Primary Health Care Center Kragujevac (decision number: 01-3344/4; approval date: 17 May 2022), and the Faculty of Medical Sciences of the University of Kragujevac (decision number: 01-13571; approval date: 24 November 2021).

Participants were divided into three groups. Group one included patients who developed gingival enlargement during amlodipine therapy (A+GE+). Group two consisted of patients with gingival enlargement of inflammatory origin who had never received amlodipine (A−GE+). Group three comprised patients treated with amlodipine who did not develop gingival enlargement (A+GE−). Patients classified with gingival enlargement of inflammatory origin were systemically healthy and were not receiving amlodipine or any other medication associated with drug-induced gingival enlargement. This group comprised individuals exhibiting clinically evident gingival enlargement with pronounced signs of gingival inflammation, as determined through clinical examination. Diagnosis relied on established clinical criteria, including alterations in gingival color, contour, and volume, as well as the presence of bleeding on probing, increased plaque accumulation, and calculus deposits. Absence of systemic disease or pharmacological exposure linked to gingival overgrowth was confirmed, supporting the classification of gingival enlargement as primarily inflammatory.

After informed consent, all participants underwent a comprehensive dental examination by the principal investigator to assess gingival enlargement and overall oral health. Periodontal status and oral hygiene were quantified using validated indices, including the Silness–Löe Plaque Index, Papilla Bleeding Index, and clinical attachment level. In the presence of gingival enlargement, the degree of enlargement was classified using the Hyperplasia Index (Angelopoulos & Goaz, modified by Pernu et al. [29]) and the Gingival Enlargement Index (Seymour et al. [30]). Also, the duration of amlodipine therapy prior to sampling was recorded for all participants and expressed as mean ± SD. After clinical examination, patients underwent surgical procedures during which gingival tissue samples were collected. These samples were subsequently used for RT-PCR analysis and histochemical evaluation.

### 4.4. Histopathological Analysis

For the histopathological assessment, samples of gingival tissue were immersed in a 10% neutral-buffered formalin solution and subsequently embedded in paraffin, forming formalin-fixed paraffin-embedded (FFPE) blocks. Thin sections, ranging from 5 to 7 μm, were prepared, deparaffinized using xylene, and rehydrated through a graduated ethanol series. These sections were then stained with Mayer’s hematoxylin for ten minutes, followed by a two-minute staining with eosin. Afterward, they underwent dehydration, clearing with xylene, and were mounted with DPX medium. To evaluate collagen content and its structural arrangement, additional sections were stained with Picrosirius Red solution, which contained 0.5 g of Direct Red 80 in 500 milliliters of saturated picric acid (Sigma-Aldrich, St. Louis, MO, USA), for one hour. Post-staining, the slides were rinsed twice in 0.005% acetic acid, dehydrated, cleared in xylene, and mounted with DPX. Prior to microscopic analysis and image capture, all slides were dried in the air for a period of 24 h.

The tissue specimens were stained with hematoxylin-eosin and Picrosirius Red staining solutions, and then photographed using an optical microscope (Leica DM2500, Wetzlar, Germany) equipped with a digital camera (Leica Flexacam i5, Wetzlar, Germany). For quantitative analysis of collagen, bright-field images of Picrosirius Red-stained sections were captured at 20× magnification, and the positive areas in visual fields were measured using ImageJ software version 1.54k (National Institute of Health, Bethesda, MD, USA). For each investigated region, we analyzed five fields per section. The results are presented as the mean value of the histological score or the mean count of the visual field (area) percentage. Fields lacking gingival tissue were excluded from further evaluation.

The pathohistological features of the samples were assessed based on several parameters, which were expressed as a cumulative score: epithelial thickness (0—normal; 1—moderate hyperplasia; 2—pronounced hyperplasia), rete ridge configuration (0—normal, 1—bulbous, 2—thin and narrow), presence of inflammation (0—absent, 1—mild, 2—moderate), and hyalinization (0—absent, 1—mild, 2—pronounced). Additionally, the inflammatory aspect was examined using the inflammation scoring system for gingival enlargement caused by amlodipine, as proposed by Siddika Selva Sume et al. [4]. This scoring system evaluated the presence and severity of inflammation in the gingival tissue and was classified as follows: 0—no signs of inflammation; 1—mild inflammation; 2—moderate inflammation; 3—severe inflammation. Histological evaluations, pathohistological scoring, and inflammatory scoring were conducted in a blinded manner by two independent observers. The agreement between raters was significant (Cohen’s kappa = 0.704 ± 0.057, 0.716 ± 0.051, respectively).

### 4.5. RT-PCR

Following surgical excision, samples of gingival tissue were promptly frozen in liquid nitrogen and stored until RNA extraction was performed. Total RNA was isolated from the tissues using TRIzol reagent (Invitrogen, Waltham, MA, USA), following the manufacturer’s instructions. The process involved phase separation with bromochloropropane, after which the aqueous phase containing RNA was precipitated with isopropanol and washed twice with 70% ethanol. The RNA pellet was then air-dried and dissolved in nuclease-free water. The concentration and purity of RNA were measured spectrophotometrically at 260/280 nm with an Eppendorf Biophotometer (Eppendorf, Hamburg, Germany). For reverse transcription, iScript Reverse Transcription Mastermix (Bio-Rad, Hercules, CA, USA) was used. Quantitative reverse transcription PCR (RT-PCR) was performed using SsoAdvanced Universal SYBR Green Supermix (Bio-Rad, Hercules, CA, USA) along with primers specific to target genes (Table 3). Housekeeping genes included β-actin. PCR amplification was conducted on an Applied Biosystems 7500 system (Applied Biosystems, Waltham, MA, USA), and relative gene expression levels were calculated via the 2^−ΔΔCt^ method (Livak & Schmittgen, 2008 [31]).

### 4.6. Statistical Analysis

Statistical analysis was performed using standard analytical methods in SPSS software (version 26.0; IBM, New York, NY, USA). Continuous variables were expressed as mean ± standard deviation or as median (interquartile range), depending on data distribution. For comparisons between two independent groups, Student’s *t*-test was used for normally distributed variables, while the Mann–Whitney U test was applied for variables with non-normal distribution. Comparisons among more than two groups were performed using one-way ANOVA for normally distributed data, whereas the Kruskal–Wallis test was used for non-normally distributed variables. Differences were considered statistically significant at *p* < 0.05. The results are presented in tables and figures.

## 5. Conclusions

This study provides further insight into the potential mechanisms underlying amlodipine-induced gingival enlargement. Although inflammatory changes were observed, they appear to represent a secondary or accompanying process rather than the primary cause of this condition. The findings suggest that amlodipine-induced gingival enlargement is likely to represent a multifactorial process in which a fibrotic component may predominate. The increased relative gene expression of α-SMA and IL-13, along with our previous results indicating molecular changes related to extracellular matrix regulation, may indicate the involvement of cytokine-mediated signaling pathways and transcriptional regulation of markers associated with fibroblast differentiation in response to the drug. In addition, the reduced MMP-1 expression observed among amlodipine-treated patients may reflect a possible direct effect of the medication on extracellular matrix turnover, favoring collagen accumulation. Overall, these results suggest that this adverse effect probably results from a complex interplay of enhanced connective tissue deposition, impaired matrix degradation, and secondary inflammation. Nevertheless, additional research with larger samples and further functional assays is needed to confirm these findings and clarify the molecular pathways involved.

### Limitations

This study is constrained by a relatively small sample size and reliance on mRNA-based analyses for selected fibrotic markers. Although this methodology offers valuable insight into transcriptional changes associated with gingival enlargement, incorporating protein-level or functional analyses could enhance the interpretation of these results. Future research involving larger patient cohorts and complementary methodological approaches is necessary to further clarify the molecular mechanisms underlying amlodipine-induced gingival enlargement.

## Figures and Tables

**Table 1:** Values of periodontal clinical indices in the studied patients.

	**A+GE+ Median (IQR)**	**A−GE+** **Median (IQR)**	**A+GE− Median (IQR)**	*p* **
Sillnes Löe Plaque Index	0.84 (0.69)	0.95 (1.13)	0.59 (1.25)	0.447
Papillae Bleeding Index *	0.50 (0.69)	1.50 (2.00)	0.16 (0.48)	0.005
Clinical Attachment Level	2.32 (3.66)	0.25 (1.12)	1.25 (0.69)	0.960
	**A+GE+ Median (IQR)**	**A−GE+** **Median (IQR)**		*p* ***
Hyperplasia Index	1.7 (1.53)	1.4 (1)		0.959
Gingival Hyperplasia Index	2.0 (0.67)	1.63 (0.65)		0.382

* *p* < 0.05; ** Mann–Whitney U test; *** Kruskal–Wallis Test.

**Table 2:** Duration of amlodipine treatment prior to tissue sampling.

	A+GE+ Mean ± SD	A+GE− Mean ± SD	*p* **
Duration (months)	14.69 ± 21.75	21.75 ± 29.798	0.440

** Independent Sample T-test.

**Table 3:** List of primers used in RT-PCR.

Primer Name	Sequence (5′-3′)
Human β-actin	F AGCACAGAGCCTCGCCTT R CATCATCCATGGTGAGCTGG
Human α- SMA	F CCGACCGAATGCAGAAGGA R ACAGAGTATTTGCGCTCCGAA
Human IL-13	F CATGGCGCTTTTGTTGACCA R AGCTGTCAGGTTGATGCTCC
Human MMP-1	F AAGGCCAGTATGCACAGCTT R TGCTTGACCCTCAGAGACCT
Human Procollagen	F CCCCCTCCCCAGCCACAAAG R TCTTGGTCGGTGGGTGACTCT

F—forward; R—reverse.

## Data Availability

The data presented in this study are available upon request.

## Abbreviations

The following abbreviations are used in this manuscript:

CCB(s)	Calcium channel blocker(s)
DIGE	Drug-induced gingival enlargement
ECM	Extracellular matrix
TGF-β1	Transforming growth factor-β1
CTGF	Connective tissue growth factor
EMT	Epithelial–mesenchymal transition
α-SMA	Alpha-smooth muscle actin
IL-13	Interleukin-13
MMP-1	Matrix metalloproteinase-1
FFPE	Formalin-fixed paraffin-embedded
